# Stress-induced pseudokinase TRB3 augments IL1β signaling by interacting with Flightless homolog 1

**DOI:** 10.1016/j.jbc.2023.104803

**Published:** 2023-05-11

**Authors:** Sumati Gonuguntla, Rohan K. Humphrey, Akshita Gorantla, Ergeng Hao, Ulupi S. Jhala

**Affiliations:** Pediatric Diabetes Research Center, University of California San Diego, La Jolla, California, USA

**Keywords:** Tribbles, TRIB3 FLII, MLK3, IKK, NFκB, iNOS, NOS2 Interleukin1, MyD88, toll-like receptor, pancreatic β cells, inflammation

## Abstract

Interleukin-1β is one of the most potent inducers of beta cell inflammation in the lead-up to type 1 diabetes. We have previously reported that IL1β-stimulated pancreatic islets from mice with genetic ablation of stress-induced pseudokinase TRB3(TRB3KO) show attenuated activation kinetics for the MAP3K MLK3 and JNK stress kinases. However, JNK signaling constitutes only a portion of the cytokine-induced inflammatory response. Here we report that TRB3KO islets also show a decrease in amplitude and duration of IL1β-induced phosphorylation of TAK1 and IKK, kinases that drive the potent NF-κB proinflammatory signaling pathway. We observed that TRB3KO islets display decreased cytokine-induced beta cell death, preceded by a decrease in select downstream NF-κB targets, including iNOS/*NOS2* (inducible nitric oxide synthase), a mediator of beta cell dysfunction and death. Thus, loss of TRB3 attenuates both pathways required for a cytokine-inducible, proapoptotic response in beta cells. In order to better understand the molecular basis of TRB3-enhanced, post-receptor IL1β signaling, we interrogated the TRB3 interactome using coimmunoprecipitation followed by mass spectrometry to identify immunomodulatory protein Flightless homolog 1 (Fli1) as a novel, TRB3-interacting protein. We show that TRB3 binds and disrupts Fli1-dependent sequestration of MyD88, thereby increasing availability of this most proximal adaptor required for IL1β receptor-dependent signaling. Fli1 sequesters MyD88 in a multiprotein complex resulting in a brake on the assembly of downstream signaling complexes. By interacting with Fli1, we propose that TRB3 lifts the brake on IL1β signaling to augment the proinflammatory response in beta cells.

Beta cell inflammation contributes to the etiology of both type 1 and type 2 diabetes (T1D and T2D). However, the rate and degree of beta cell inflammation in T1D *versus* T2D are distinctly different. The strength and nature of signal-dependent physiologic responses are determined by spatial and temporal dynamics of signal activation (1). In turn, the dynamics of signaling are strongly influenced by the scaffolds and adaptors that molecularly wire the signaling machinery (2), highlighting the need for a more nuanced understanding of proinflammatory signaling in the beta cell. TRB3 is a stress-induced pseudokinase whose function is unmistakably associated with a number of pathologies, including obesity, diabetes, and Parkinson’s disease (3, 4). At the cellular level, TRB3 has been characterized as an inducer of endoplasmic reticulum (ER) stress, insulin resistance, and apoptosis (3, 5, 6). However, the molecular mechanism/s by which TRB3 exerts its effects are less well understood. The gap in information is partly because TRB3 is a pseudokinase that lacks critical motifs required for ATP binding and transfer (7), resulting in absence of a clear readout of TRB3 molecular action. We have previously reported that, in beta cells, IL1β rapidly induces phosphorylation of MAP3K MLK3 to, in turn, stimulate JNK stress kinase. pMLK3 also binds and stabilizes TRB3 protein (5) leading to a feed forward mutually stabilizing effect on components of the JNK module including JIP1, MLK3, and TRB3 (8). Inhibition of MLK3 protects beta cells from caspase-dependent cell death in the early phases of inflammation but has no effect on the activation of the IKK-NFκB pathway (5), a second signaling arm that is required for a full and robust inflammatory program. Here we provide evidence to show that, in addition to MLK3-JNK signaling, TRB3 also regulates beta cell inflammation by attenuating TAK1-IKK phosphorylation and subsequent NFκB-mediated expression of key genes that drive beta cell inflammation. Although TRB3 has been reported to function as an allosteric regulator of multiple kinases (3, 9, 10), in our assay, we were unable to identify clear and direct binary interactions between TRB3 and kinases that regulate the IKK-NFκB pathway in the beta cell. Here we describe a novel interaction between TRB3 and the LRR (leucine-rich repeat) protein Flightless Homolog 1 (Fli1). Fli1 is an actin-binding molecular scaffold protein (11, 12), which can cooperatively enhance or competitively inhibit cellular inflammation (13). TRB3 engages Fli1 in response to IL1β treatment of the beta cell and functions as a scaffold protein that can fine-tune kinase activation for stimulus-dependent activation of the inflammatory response.

## Results

### TRB3 is required for optimal IL1β-mediated signaling

**Figure 1:**
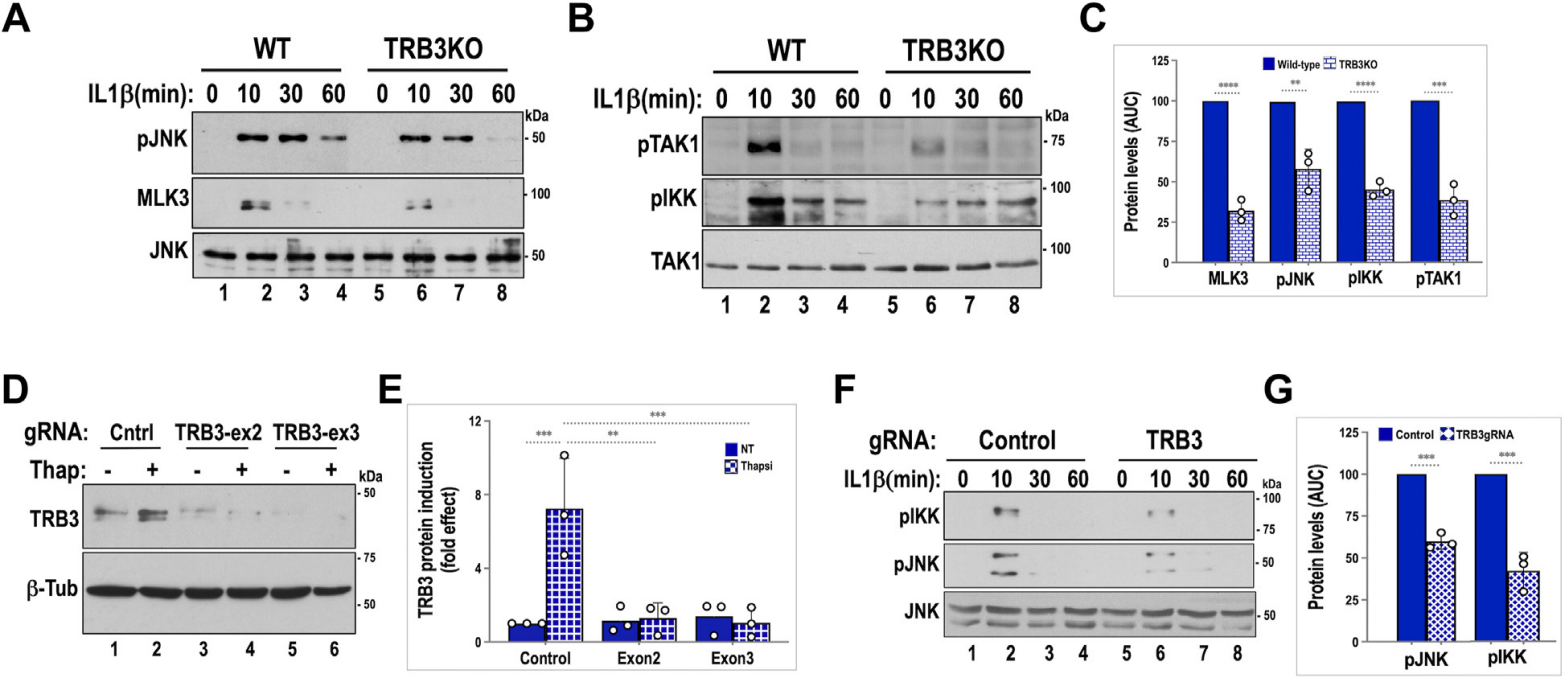
**Loss of TRB3 leads to attenuated activation of JNK and IKK in islets and Min6 cells.***A*, wildtype (lanes 1–4) and TRB3KO (lanes 5–8) islets were treated with 20 ng/ml of IL-1β for the indicated times, and lysates were subjected to Western blotting using anti-MLK3 and phospho-JNK antibodies. Total JNK protein levels are shown. *B*, wildtype (lanes 1–4) and TRB3KO (lanes 5–8) islets were treated with 20 ng/ml of IL1β for the indicated times, and lysates were subjected to Western blotting using anti-phosphoTAK1 and phospho-IKK antibodies. Total TAK1 protein levels are shown. *C*, densitometric analyses of time-dependent JNK, IKK, and upstream MAP3Ks MLK3 and TAK1 were graphed, and total kinase induction/activity over time was assessed by calculating area under the curve (AUC). Average values from three independent time course experiments are presented as percent fold effects of control-WT samples. Two-tailed unpaired t-test was performed to compare WT and TRB3KO (∗∗∗∗*p* < 0.0001, ∗∗∗*p* < 0.0005, ∗∗*p* < 0.05). *D*, efficacy of TRB3 knockdown was tested using Min6 cells electroporated with control (lanes 1–2) or gRNA constructs targeted to exon 2 (lanes 3–4) or exon 3 (lanes 5–6) of the mouse TRB3 gene. TRB3 expression was induced using thapsigargin (400 nM for 5 h) as shown. *E*, quantification of TRB3 protein levels from Figure 1*D* represents an average of three independent experiments (∗∗∗*p* < 0.001, ∗∗*p* < 0.005). *F*, Min6 cells electroporated with control (lanes 1–4) or TRB3 gRNA (lanes 5–8) were treated with 20 ng/ml IL1β for the indicated times, and lysates were subjected to Western blotting using anti-phospho-IKK and phospho-JNK antibodies. Total JNK protein levels are shown. *G*, graph shows average, IL-1β-induced kinase activation from TRB3 gRNA-expressing cells. Data presented are calculated and expressed as percent AUC of control within each of three independent experiments (Two-tailed unpaired t-test showed a significant difference of using TRB3 gRNA. ∗∗*p* <0.005). Unless specified, data presented in bar graphs show average fold effects from three independent experiments, calculated by normalizing all values to WT control. In each case, mean values ± SD of three experiments are presented.

The pseudokinase TRB3 is induced by multiple stress stimuli including cytokines and agents that induce ER stress (5, 6). In the beta cell, MAP3K-JNK and IKK-NFκB signaling pathways are the most potent mediators of cytokine-dependent inflammation and ensuing decline in beta cell survival (14, 15). We have previously reported that, in beta cells, the pseudokinase TRB3 modulates the strength of JNK activation by stabilizing a cytokine-activated signaling module consisting of MLK3, MKK7, JIP1, and JNK1/2 (5, 10). As previously reported (10), and shown here in Figure 1*A*, such a role for TRB3 is associated with optimal activation of MLK3 and sustained activation of JNK in islets from WT mice, and both are attenuated in islets from TRB3KO mice. These results prompted us to examine whether TRB3 could similarly modulate activation of the IKK-NFκB pathway. IL1β stimulation of the canonical IKK-NFκB pathway requires the activation of TAK1 kinase to activate downstream gene expression (16, 17). MLK3 and TAK1 are both activated by signal-dependent ubiquitination and autophosphorylation, downstream of the IL1β receptor (16, 18, 19). We therefore examined IL1β-mediated phosphorylation of TAK1 and IKK in islets from WT and TRB3KO mice. Isolated islets from WT TRB3KO mice were stimulated with IL1β in a time-dependent manner, and islet extracts were used for Western blotting. As seen in Figure 1, *B* and *C*, when compared with WT islets, TRB3KO islets showed 50 to 60% lower induction of both TAK1 and its downstream target IKK.

Next, we examined whether short-term loss of TRB3 could similarly impact activation of IKK and JNK in Min6 insulinoma cells. For knockdown of TRB3, we used the CRISPR-Cas9-Nickase system to target two independent guide RNAs, directed to two different exons of nascent TRB3 mRNA. gRNA design to suppress TRB3 induction is shown in Fig. S1. Nucleofection of Min6 cells showed the efficacy with which each gRNA suppressed stress-induced expression of TRB3 protein in Min6 cells (Fig. 1, *D* and *E*). Using TRB3 targeted gRNA, we observed an attenuation of IL1β-dependent JNK and IKK activation in Min6 cells. These results closely parallel the results obtained from TRB3KO islets. Total JNK was used as loading control (Fig. 1, *F* and *G*).

### Loss of TRB3 attenuates NFκB-dependent gene activation

**Figure 2:**
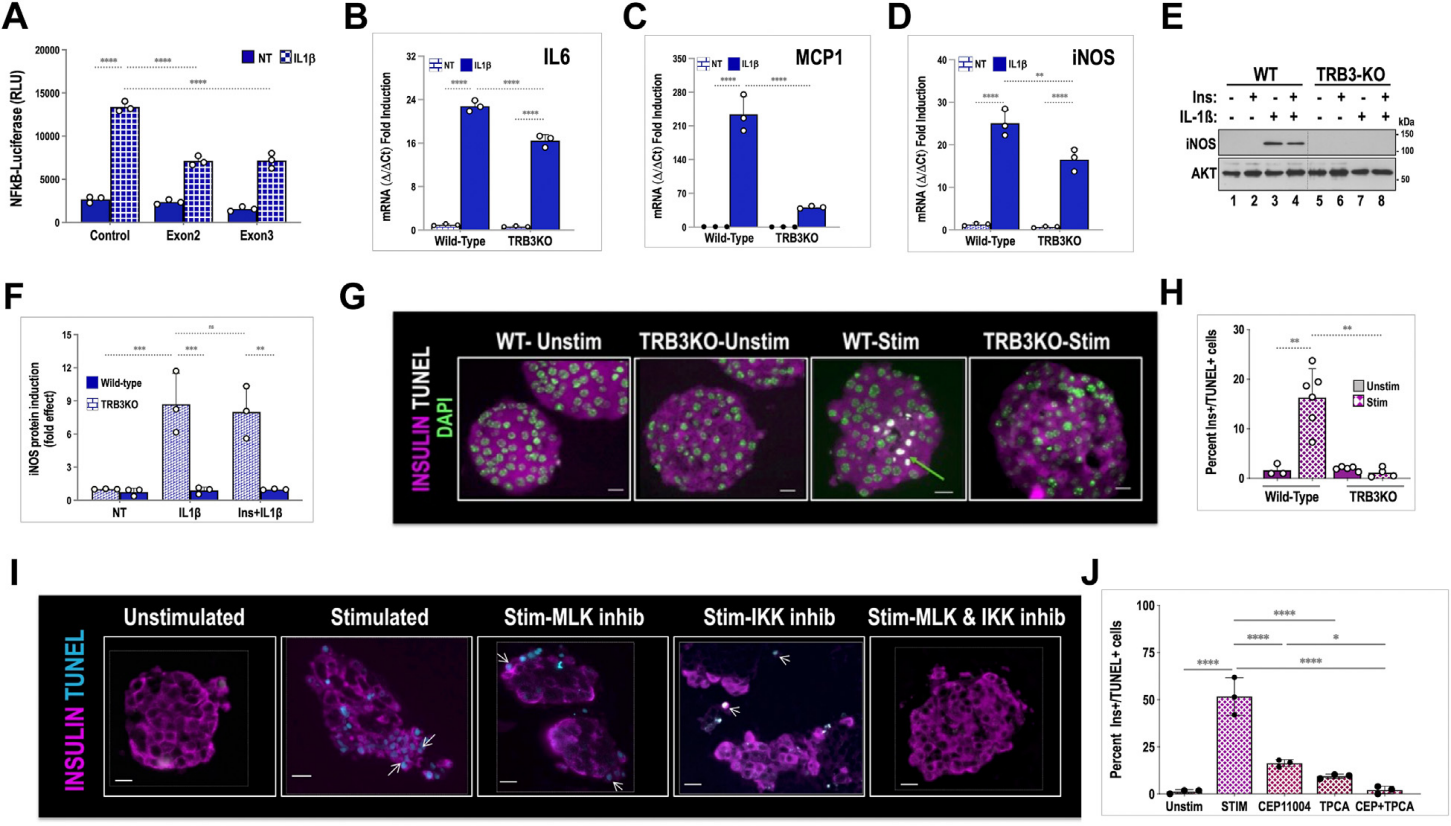
**TRB3 is required for optimal activation of NFκB target genes.***A*, luciferase reporter assay from Min6 insulinoma cells transiently transfected with firefly NFκB-Luciferase reporter in the presence of control gRNA or TRB3 gRNAs. Cells were treated with 20 ng/ml IL-1β (*solid bars*) or *left* untreated (*hatched bars*). Data show results from one of three representative transient transfection experiments. Two-way ANOVA was performed for TRB3 gRNAs against control (*∗∗∗∗*p < 0.0001 ). *B*–*D*, relative mRNA expression from IL1β-treated primary islets for IL6 (3 h), MCP1/CCL2 (6 h), and iNOS (3 h) gene expression, respectively, using RNA isolated from WT and TRB3KO mice. Two-way ANOVA was performed to compare WT and TRB3KO (*IL6* and *MCP1*∗∗∗∗*p* < 0.0001, for iNOS, ∗∗*p* < 0.004). Data represent an average of fold mRNA induction from three independent experiments each performed using mRNA from pooled islets of control and TRB3KO mice. *E*, Western blot examining iNOS expression in protein extracts derived from islets of WT (lanes 1–4) and TRB3KO (lanes 5–8) mice, treated with 20 ng/ml IL-1β and/or 100 nM insulin for 8 h as indicated. Insulin stimulation preceded IL-1β treatment by 10 min. *F*, quantification of iNOS protein was performed using densitometric analysis of three independent experiments and represents average protein induction expressed as a fold effect of WT control islets. (∗∗∗*p* < 0.004, ∗∗*p* < 0.001). *G*, islets from WT and TRB3KO mice were cocultured with immune-activated splenocytes from WT mice for 24 h and processed as described. Insulin stain was pseudocolored magenta, TUNEL staining is pseudocolored white and indicated by an arrow. Nuclei were counterstained with DAPI. The scale bar represents 20 μm. *H*, quantification of insulin/TUNEL double+ cells expressed as a % of total, insulin-expressing cells from multiple islets using sections from three independent experiments. (∗∗*p* < 0.01). Note: Datasets used for statistical analysis in Figure 2*F* have been previously published (10). *I*, human donor islets were cocultured with unstimulated or stimulated/activated human splenocytes as described (18). Prior to coculture, islets were pretreated for 1 h with MLK inhibitor CEP11004 (500 nM) or IKK inhibitor TPCA1 (5 μM) as indicated. The islets were harvested and fixed after 30 h, sectioned, and processed for TUNEL staining and immunofluorescence using anti-insulin antibodies. For clarity, insulin staining is pseudocolored magenta and TUNEL staining pseudocolored cyan. White arrow shows apoptotic cells. The scale bar represents 20 μm. *J*, quantification of insulin-expressing cells positive for TUNEL staining, expressed as a % of total insulin+ cells. TUNEL staining was performed simultaneously using sections from four independent coculture experiments. Data were analyzed using one-way ANOVA (∗∗∗∗*p* < 0.0001, ∗∗*p* < 0.005, ∗*p* < 0.05).

Studies have demonstrated that activation of IKK and its downstream effector NFκB is crucial for cytokine-induced inflammatory damage of beta cells (20, 21, 22). We examined whether TRB3 expression is required for optimal NFκB-dependent gene activation. Using an NFκB response element–driven luciferase reporter in transient transfections, we examined whether coexpression of TRB3 gRNA impacted reporter activity in IL1β-stimulated Min6 cells. As seen in Figure 2*A*, IL1β treatment resulted in a 10- to 12-fold induction of NFκB-luc activation in the presence of gRNA control, while presence of TRB3 gRNA attenuated IL1β-dependent NFκB-luc activation, dropping by roughly 60% compared with control. These data indicated that the attenuation of IKK seen in TRB3-deficient islets and Min6 cells extends to downstream NFκB-dependent gene expression. A reporter construct driven by multimerized NFκB response elements does not reflect the complexity of promoters that drive endogenous genes, where additional transcriptional activators could reduce/enhance the impact of IKK activation on gene expression. We examined whether loss of TRB3 impacted mRNA expression of known NFκB targets previously implicated in the inflammatory process. Isolated islets pooled from multiple WT or TRB3KO mice were treated with IL1β as indicated. GAPDH mRNA was used as an unchanging control. Interestingly, loss of TRB3 did not uniformly impact expression of NFκB target genes. Compared with WT islets, IL1β treatment of TRB3KO islets revealed a small but significant decrease (30%) in IL1β-stimulated expression of IL6 mRNA in TRB3KO islets (Fig. 2*B*). Similarly upon IL1β treatment, mRNA for iNOS (inducible nitric oxide synthase-NOS2) gene was reduced in TRB3KO islets by 30 to 40% (Fig. 2*D*). By contrast, compared with WT islets, macrophage chemoattractant protein MCP1(CCL2) was strongly downregulated (80%) upon IL1β treatment of TRB3KO islets (Fig. 2*C*). No impact was seen on mRNA of IL1β, and TNF-α in these islets (not shown) at any time point. WT islets showed high basal expression of Cycloxygenase-2 (COX2) mRNA, which trended lower approximately 20% in TRB3KO islets (not shown). Overall, our results suggest that partial/attenuated IKK activity seen in TRB3KO appears to have a differential effect on downstream NFκB-driven target gene expression.

### Islets of TRB3KO mice fail to show IL1β-induced expression of iNOS protein

Although mRNA levels are an important indicator for understanding the role of genes that drive signal-dependent cellular behavior, biological effects are determined by protein expression. Interestingly, gene expression profiles are not predictive of a corresponding change in protein expression (23). iNOS protein has been shown to play a pivotal role in cytokine-induced beta cell death (24), prompting us to examine whether reduced iNOS gene expression in TRB3KO islets resulted in decreased protein expression. We found that post cytokine treatment, iNOS protein is maximally induced by 8 h, persisting up to 12 to 15 h (not shown). We therefore treated pooled islets from each group with IL1β for 8 h, and as shown in Figure 2*E*, Western blotting of islet extracts showed a robust induction of iNOS in WT islets, which was almost entirely abolished in TRB3KO islets. Repression of iNOS protein in TRB3KO islets is much stronger than the effect noted for iNOS mRNA suggesting additional roles for TRB3 in iNOS protein expression. The proapoptotic role of TRB3 in many cell types has been attributed to TRB3-dependent inhibition of AKT prosurvival kinase (3). Studies in primary hepatocytes have revealed that AKT activity lowers iNOS expression either directly or indirectly *via* inhibition of GSK3 (25, 26). We therefore examined whether the steep decrease in iNOS protein expression in TRBKO islets could be overcome by pretreatment of insulin. We found that WT islets pretreated with insulin showed a marginal but statistically insignificant decrease in iNOS expression (Fig. 2, *E* and *F*). These data clearly show that the proinflammatory function of TRB3 extends well beyond any ability to inhibit AKT in beta cells. Taken together our data show that TRB3 is required for optimal IL1β-induced expression of iNOS gene, a crucial contributor to cytokine-activated oxidative stress and acceleration of beta cell death (27).

### Genetic ablation of TRB3 confers resistance to cytokine-induced beta cell death

IKK-NFκB and JNK stress kinase constitute the most potent proinflammatory pathways. Given that TRB3 augments both pathways, in an experiment identical to that reported in our previous study (9), we examined whether loss of TRB3 could exert a protective effect on cytokine-induced cell death. We improvised and developed an islet culture method that could induce beta cell death at levels observed in T1D (5) (see schematic in Fig. S2). Splenocytes were activated to elicit a robust immune reaction (see Table S1*A*) using anti-CD3 and anti-CD28 antibodies, while unstimulated splenocytes served as a control. Isolated islets from both TRB3KO and WT mice were suspended in transwells and incubated with stimulated splenocytes from WT mice, and after 30 h, they were harvested to examine markers of apoptosis. Compared with WT islets, upon coculture with stimulated splenocytes, TRB3KO islets were largely negative for TUNEL staining (Fig. 2, *G* and *H*). These data show that TRB3 expression is required to augment proinflammatory signaling to thresholds high enough for triggering cytokine-stimulated cell death.

### Pharmacologic inhibition of MLK3 and IKK suppresses cytokine-induced beta cell apoptosis

TRB3KO islets show attenuated expression of two critical pathways in cytokine-induced islet inflammation. We have previously shown that the MLK3-JNK pathway is activated in the early stages of cytokine-induced beta cell death and pharmacologic inhibition of MLK family of kinases (500 nM CEP11004) delays onset of beta cell death by several hours. The JNK stress kinase pathway has been associated with mitochondrial outer membrane permeabilization *via* mitochondrial translocation of proapoptotic BCL2-family protein BAX (28). Notably, MLK3 inhibition had no effect on IKK-NFκB-dependent iNOS activation in islets (5). Given that TRB3KO islets display attenuation of both MLK3-JNK and TAK1-IKK pathways, and that IKK-NFκB targets are instrumental in inducing islet dysfunction, we examined whether pharmacologic inhibition of IKK-NFκB activation could supplement the effect of MLK3 inhibition. We also examined whether inhibition of both pathways could inhibit rather than just delay progression of beta cell apoptosis. Although pharmacologic inhibition of kinases is often fraught with nonspecific inhibition of additional pathways, the main goal of these experiments was limited and meant solely to demonstrate proof of principle that the MLK3-JNK and TAK1-IKK pathways are together necessary and sufficient to induce apoptosis in primary islets.

We used human donor islets and cadaveric human spleens in this assay as described (18). Primary donor human islets were suspended in transwells and cocultured with plated activated human splenocytes for approximately 30 h. Splenocytes were activated to elicit a robust immune reaction (see Table S1*B*) using anti-CD3 and anti-CD28 antibodies. Again, unstimulated splenocytes served as a control. As shown in Figure 2*I*, we examined whether inhibition of MLK3 using CEP11004 along with IKK inhibitor TPCA1 was sufficient to rescue beta cells from cytokine-activated cell death. A 30-h incubation of islets with stimulated splenocytes resulted in widespread cell death (approximately 40%) as judged by strong TUNEL staining, which detects DNA strand breaks associated with apoptosis. Islets incubated with stimulated medium also displayed extensive loss of islet morphology and noticeable loss of insulin from beta cells across islets, which is distinctly different from results seen with mouse islets. Pretreatment of islets with MLK3 inhibitor CEP11004 or TPCA1, a powerful IKK inhibitor, showed that they were each partially effective at inhibiting widespread cell death (reduced from roughly 40–50% without drugs to approximately 8–15% (Fig. 2*J*) within the time frame of this study). When CEP11004 and TPCA1 were both used, we observed near-complete inhibition of cell death, a better retention of insulin, and preservation of islet morphology. It may be noted that BAY-117085, an alternate inhibitor of the TAK1-IKK pathway, had a comparable effect on cell death (not shown). Although we cannot completely rule out the off-target effects of each drug, our data show that inhibition of both MLK3-JNK and TAK1-IKK pathways could be sufficient to preserve human beta cell integrity in response to inflammation. These results are consistent with a cytokine-induced, proinflammatory and proapoptotic role for TRB3 in beta cells.

### Cytokines enhance TRB3 interaction with the scaffold protein Flightless homolog I

**Figure 3:**
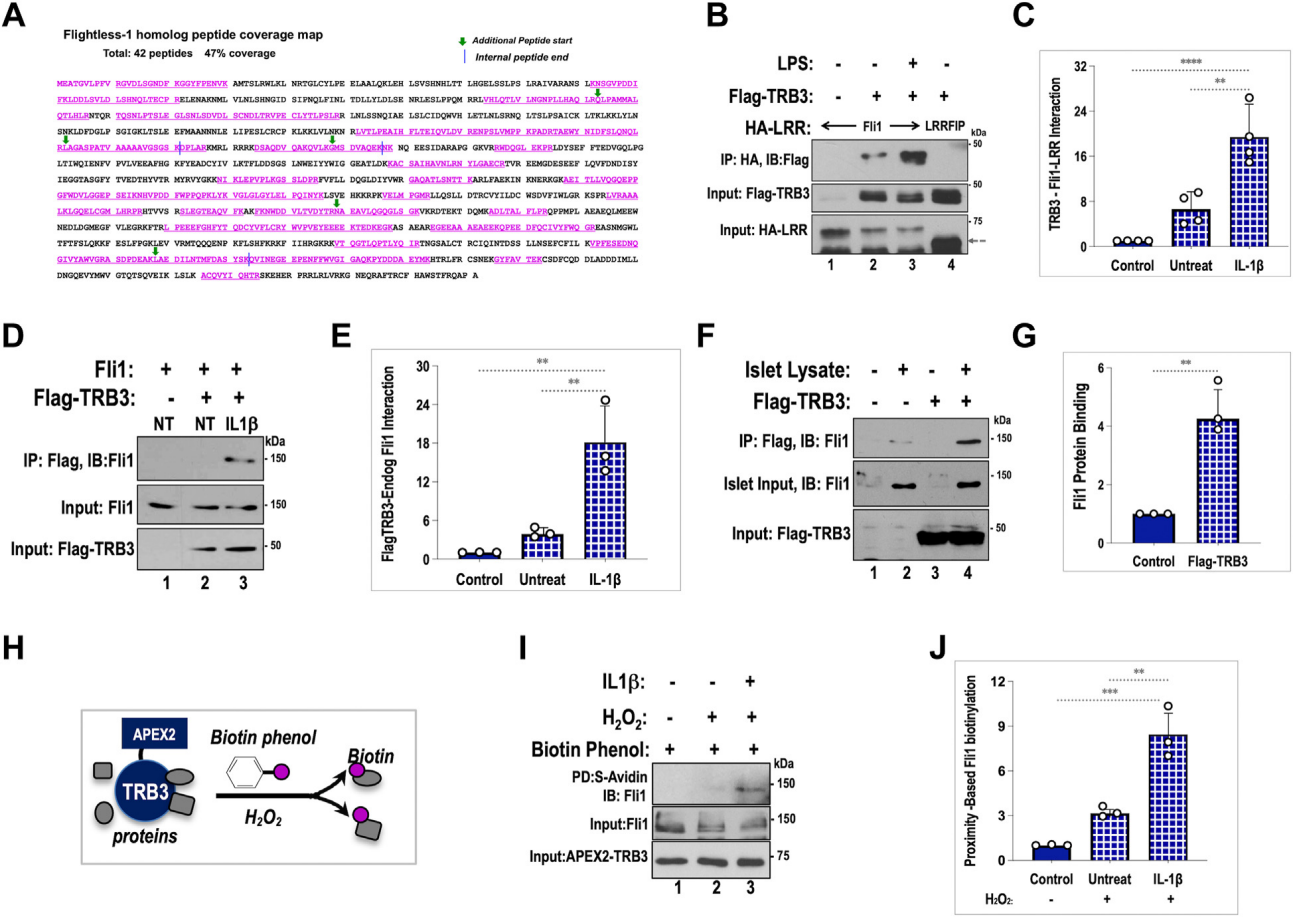
**Cytokines induce TRB3 interaction with Fli1 (Flightless Homolog 1).***A*, identification of Flightless-homolog 1 as a TRB3-interacting protein using mass spectrometry. The peptides identified are highlighted in *pink* font. Overlapping peptides are indicated with a *green arrow* for start site and *blue line* denoting end of the internal peptide. Only peptides with peak intensity ratio of 2-fold or higher on a Log2 scale were considered. *B*, coimmunoprecipitation assay examines interaction between Fli1 and TRB3 proteins using the HA-tagged LRR domain of Fli1 and Flag-TRB3, coexpressed in HepG2 cells, without (lane 2) and following 30-min treatment with LPS (10 ng/ml) (lane 3). HA-Fli1-LRR in the absence of TRB3 served as control (lane 1). Note: TRB3 does not interact with the HA-LRR-domain of LRRFIP2 (lane 4). *D*, coimmunoprecipitation assay examines interaction between Flag-TRB3 and endogenous Fli1 from Min6 cells clustered into pseudoislets, without (lane 2) and upon 30-min treatment with ILβ (10 ng/ml) (lane 3). *F*, protein interaction between purified Flag-TRB3 (lanes 3, 4) incubated with lysates from human islets (lanes 2, 4) showing potential for TRB3-Fli1 in human islets. *C*, *E*, and *G*, quantitative analysis of data from the Figure, *B*, *D*, and *F* depicted as fold induction of TRB3–Fli1 interaction over control. In each case, graphs represent average, fold induction of TRB3-Fli1 interactions from three independent experiments each normalized to value of WT control. (For *C*, ∗∗∗∗*p* < 0.0001, ∗∗*p* < 0.001, and *E* and *G*, ∗∗*p* < 0.005). *H*, schematic of proximity-induced biotinylation of APEX2 (ascorbic acid peroxidase)-TRB3 interacting proteins. Peroxidase activity of APEX2-TRB3 fusion can cleave Biotin-phenol in the presence of 0.5 mM H_2_O_2_ resulting in spontaneous biotinylation of proteins within 20 nm of TRB3. *I*, APEX2-TRB3-expressing Min6 cells were treated with Biotin-phenol (2 h) followed by 1 min of H_2_O_2_ treatment. IL1β stimulation was for 30 min prior to peroxide treatment (lane 3). Streptavidin-agarose was used to pull down biotinylated proteins and examined for presence of Fli1 using Western blotting. Biotin-phenol without peroxide treatment was used as a control (lane 1). *J*, Fli1 recovered in Western blots of streptavidin pulldowns was quantified and presented in graph format. Again, the data are represented as average fold difference of Fli1 recovered by streptavidin beads in three independent experiments (∗∗*p* < 0.001, ∗∗∗*p* < 0.0005).

TRB3 structurally resembles kinases but lacks critical residues for catalytic phosphotransfer. Therefore, it mediates its actions by allosterically modulating the function of interacting proteins. The apoptotic potential of TRB3 has been attributed to its ability to interact with and inhibit AKT, a potent prosurvival and progrowth kinase (3). We have shown that insulin pretreatment (a stand in for AKT activation) had marginal effects on the expression of damage-inducing iNOS protein, suggesting that TRB3 proinflammatory action likely involves additional cellular mechanisms. In order to get a better understanding of the mechanism by which TRB3 augments IL1β-induced IKK-iNOS activation, we examined whether TRB3 interacts with proteins that could augment cytokine/IKK signaling. We interrogated the TRB3 interactome by expressing Flag-TRB3 in Min6 mouse insulinoma cells followed by standard coimmunoprecipitation (co-IP) assays and proteolytic (tryptic) digestion of immunoprecipitates followed by mass spectrometry (see Experimental procedures). The experiment was repeated two more times using identical conditions. In the absence of any direct modulator of IKK activation/signaling in the interactome (see Table S1), we focused on two LRR family proteins, shown to previously regulate lipopolysaccharide (LPS) signaling in immune cells. The two proteins, Fli1 (Flightless homolog 1) and LRRFIP2 (leucine-rich repeat Flightless interacting protein), are scaffold proteins that function by modulating availability of MyD88, the most proximal adaptor in IL1β as well as LPS-TLR4 signaling cascade (29, 30). Shown in Figure 3*A* is the protein sequence of murine Fli1, with recovered peptides highlighted in color. In a representative mass spectrometry analysis of TRB3 immunoprecipitates, we recovered 42 unique peptides that mapped to Fli1 protein and displayed a 2-fold (log-scale) or higher recovery in the presence of TRB3 *versus* control. The peptides together covered 47% of the 1261 amino acids of Fli1 protein. The protein itself consists of multiple repeats of two independent domains, including the aforementioned LRR in the N terminus and the actin-binding gelsolin domain in the C terminus (11). LRR motifs consisting of 20 to 30 amino acids are present in a wide variety of proteins; their distinct shape make them versatile motifs for protein interactions (31). We examined whether the LRR domain of Fli1 (HA-tagged Fli1 N-terminal 400 amino acids) could immunoprecipitate Flag-TRB3 expressed in HepG2 cells. Using a co-IP assay followed by Western blotting, we found that HA-Fli1-LRR could indeed pull down coexpressed Flag-TRB3. Fli1 has been shown disrupt postreceptor LPS signaling by inhibiting the docking of MyD88 to the intracellular domain of TLR4 (29, 30). Given the importance of TRB3 in augmenting inflammation, we examined whether LPS could enhance TRB3–Fli1 interaction. Indeed, the presence of LPS strongly induced TRB3–Fli1 interaction, even as total levels of HA-Fli1-LRR were decreased upon LPS treatment (Fig. 3, *B*, and *C*). We also examined if the LRR domain of HA-LRRFIP2 could immunoprecipitate Flag-TRB3, but we saw no interaction between the proteins (Fig. 3*B*). These results suggest that TRB3 interaction with Fli1 is likely to be specific and not a result of generic interaction with proteins bearing LRR motifs. These data also suggest that interaction with LRRFIP2 observed in the interactome maybe indirect and potentially *via* Fli1.

IL1β is one of the strongest inducers of beta cell inflammation, and similar to LPS-TLR4 signaling, IL1β stimulation leads to MyD88 recruitment and docking at the intracellular domain of the IL1β receptor. We therefore examined whether TRB3 could immunoprecipitate endogenous Fli1 and whether this interaction could be augmented by IL1β. Flag-TRB3 was expressed in Min6 cells, the cells treated with IL1β, 30 min prior to harvesting. We performed these experiments using TRB3-expressing Min6 cells that were clustered/aggregated to form pseudoislets, because an actin-binding scaffold protein like Fli1 maybe is more stably engaged in clusters. As shown in Figure 3*D*, Western blotting of Flag immunoprecipitates under these conditions did not show appreciable interaction between TRB3 and endogenous Fli1 in untreated cells. However, pretreatment with IL1β enabled and significantly increased Flag-TRB3 interaction with endogenous Fli1 (Fig. 3*E*). We also examined if TRB3 can interact with Fli1 in human islet extracts. We expressed and purified Flag-TRB3 from 293T cells, followed by purification using anti-Flag antibody-linked agarose beads. The Flag-TRB3 immunoprecipitates were washed under high salt conditions to remove TRB3-bound endogenous Fli1 from 293T cells before incubation with human islet protein extracts. Flag-antibody-cross-linked agarose beads were used as control. As seen in Figure 3*F*, TRB3 does interact with Fli1 present in human islet extracts suggesting a potentially similar role for TRB3 in human islets. Data shown in Figure 3*G* represent an average of three independent experiments.

Native, nondenaturing conditions such as those used to examine TRB3-interacting proteins in this study can result in co-IP of direct protein interactors as well associated proteins in larger complexes not directly in contact with TRB3. We therefore assessed whether Fli1 protein interacts directly with TRB3 *in situ*. We used an assay that enabled proximity-dependent biotinylation of TRB3-interacting proteins. Briefly, this assay is premised on the fact that, in the presence of H_2_O_2,_ ascorbic acid peroxidase (APEX2) can activate biotin radicals using biotin phenol as substrate (see schematic Fig. 3*H*). The short-lived biotin radicals thus generated are estimated to spontaneously attach to proteins within a range of 2 to 10 nm, which is about the width of an average protein. Within such a short range, directly interacting proteins are most likely to be biotinylated. Based on this reasoning we examined whether APEX2-TRB3 fusion protein could biotinylate endogenous Fli1. The results of this assay are shown in Figure 3*I*. Cells expressing APEX2-TRB3 were treated with biotin-phenol, 1 min pulse of H_2_O_2_, or both, followed by streptavidin pulldown of biotinylated proteins. Notably, streptavidin pulldowns showed the presence of Fli1 protein only in the presence of both biotin-phenol and H_2_O_2._ Furthermore, TRB3–Fli1 interaction was significantly enhanced in the presence of IL1β (Fig. 3, *I* and *J*). When taken together, data from Figure 3 show that TRB3 verifiably interacts with Fli1 protein and use of a variety of approaches provides confidence in the authenticity of TRB3–Fli1 interaction.

### TRB3 disrupts Fli1–MyD88 interaction

**Figure 4:**
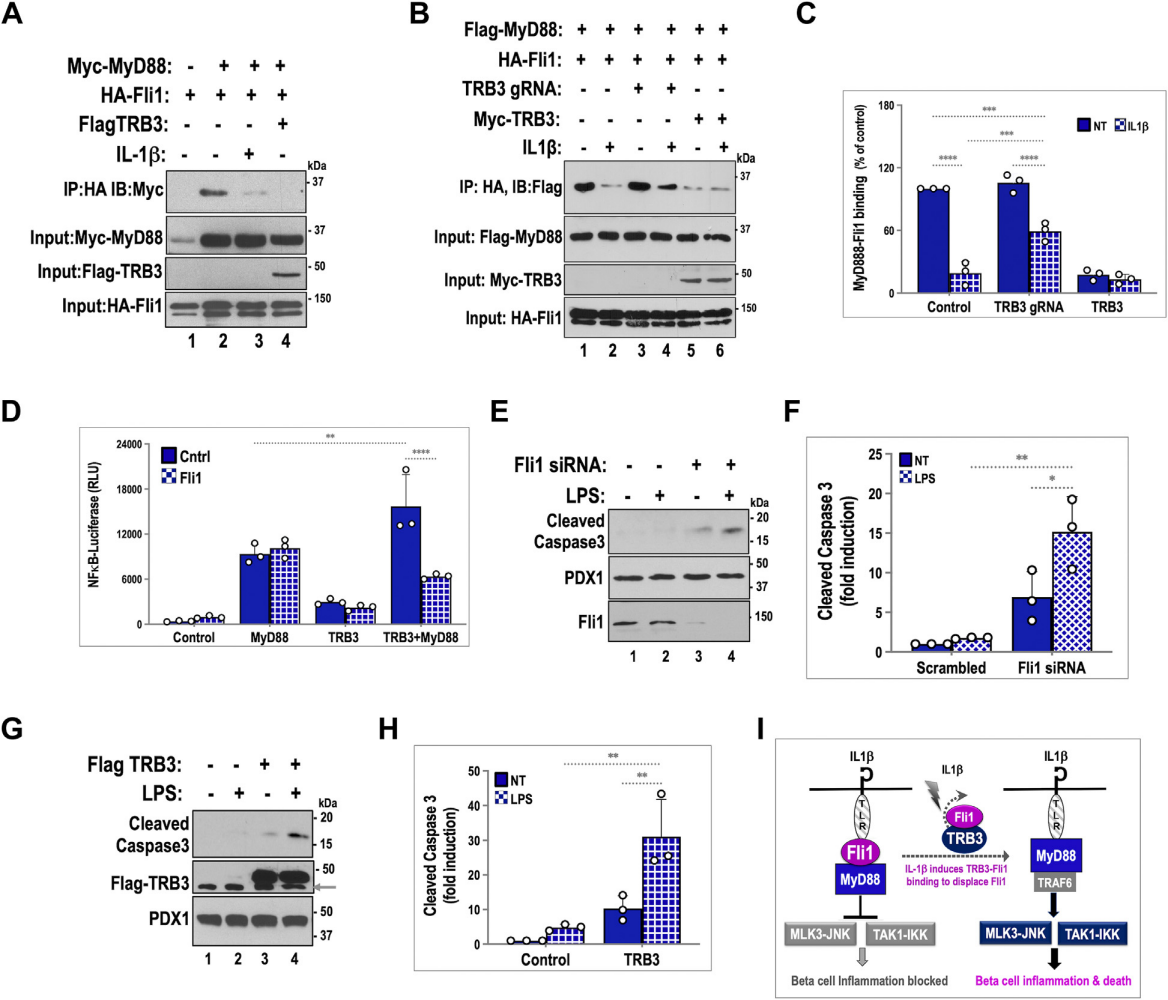
**TRB3 disrupts Fli1-dependent anti-inflammatory sequestration of MyD88.***A*, protein interaction between Fli1 and MyD88 proteins was assessed using lysates from Min6 cells transfected with HA-Fli1 (1–4) and Myc-MyD88 (lanes 2–4) in the presence of control vector (lane 1), upon 30 min IL1β (10 ng/ml) treatment (lane 3), or expression of HA-TRB3 (lane 4). Inputs for each protein are shown. *B*, impact of TRB3 knockdown on Fli1 and MyD88 protein interaction was assessed using identical conditions as in Figure 4*A*, in the presence of control gRNA, (lanes 1–2), TRB3 gRNA (lanes 3–4) and Myc-TRB3 (lanes 5–6), and in the presence of vehicle (lanes 1, 3, and 5) or upon 30-min pretreatment with IL1β. Of note, TRB3 knockdown results in partial retention of Fli1–MyD88 interaction even in the presence of IL1β (compare lanes 2 and 4). *C*, Fli1–MyD88 interactions from Figure 4*B* were quantified, and the amount of MyD88 recovered was expressed as a fraction of MyD88 input. Stippled and *solid bars* denote Fli1–MyD88 binding in the absence or presence of IL1β, respectively. Two-way ANOVA was performed to compare WT and TRB3-gRNA (∗∗∗*p* < 0.0005, ∗∗∗∗*p* < 0.0001). *D*, luciferase reporter assay from Min6 insulinoma cells transiently transfected with NFκB-Luciferase (firefly) reporter in the presence of MyD88, TRB3, or both, in the absence (*solid bars*) or presence (*stippled bars*) of Fli1. Data presented are an average of triplicate values ± SD from a representative experiment (n = 5). Two-way ANOVA of TRB3 expression on MyD88 effect (∗∗*p* < 0.005, ∗∗∗∗*p* = 0.0001). *E*, LPS treatment 10 ng/ml (lanes 2 and 4) enhances Fli1-siRNA (lanes 3–4) knockdown-mediated cell death as judged by increasing expression of cleaved caspase 3 (compare lanes 3 and 4). LPS treatment alone (lane 2) does not induce cell death in the presence of scrambled siRNA (lanes 1–2). *G*, adenovirus-mediated TRB3 overexpression (72 h, lanes 3–4) induces low-level but nonsignificant cell death (lane 3), which is enhanced by 48-h LPS treatment (10 ng/ml). LPS treatment alone (lane 2) does not induce cell death/caspase 3 cleavage in the presence of control virus (lanes 1–2). *F* and *H*, fold induction of cleaved caspase 3 was calculated using densitometric analysis of data from three independent experiments shown in Figure 4, *F* and *H*, respectively, and analyzed using two-way ANOVA (For *F*, ∗∗*p* < 0.005, and ∗*p* < 0.05, and for *H*, ∗∗*p* < 0.01). *I*, schematic model summarizing data presented and depicting mechanism by which TRB3 engages Fli1 to impact IL1β signaling.

In order for protein–protein interactions to be valid, they must satisfy appropriate functional parameters and be consistent with the biological roles for each of the interacting proteins. Upon examination of the literature, we found that Fli1 has the ability to regulate TLR4 signaling by binding and sequestering MyD88, a proximal and primary adaptor that recruits the downstream protein complexes required for IL1β-TLR4 signaling (30). It was reported that, upon LPS stimulation, Fli1 dissociates from MyD88, freeing up MyD88 to participate in TLR4 signaling. Given that TLR4 and IL1β receptor both recruit MyD88 as the first adaptor (32), we examined whether MyD88–Fli1 interaction was impacted by TRB3 or by IL1β. To this end, we again used tagged proteins expressed in transfected Min6 cells, followed by co-IP assays. As seen in Figure 4*A*, under basal conditions, HA-Fli1 successfully immunoprecipitated Myc-MyD88. Similar to LPS (29), a short 30-min treatment with IL1β also dramatically reduced HA-Fli1 pulldown of Myc-MyD88. Notably, coexpression of TRB3 markedly decreased Fli1–MyD88 interaction as well. These results suggest that TRB3 mimics IL1β-stimulated, postreceptor dissociation of Fli1 from MyD88. We have previously shown that TRB3 protein is nearly undetectable under basal conditions but is stabilized and detectable within 20 to 30 min following islet/Min6 cell stimulation with IL1β (5). This raises the intriguing possibility that IL1β-stimulated TRB3 induction could augment and prolong IL1β signaling by preventing premature reassociation of MyD88 with the inhibitory Fli1 protein. If true, then the absence of TRB3 should provide a more permissive environment for Fli1–MyD88 interaction even after IL1β stimulation. We examined this question by using Fli1–MyD88 co-IP assays similar to those in Figure 4*A*, this time using an excess of TRB3 or knockdown of TRB3 expression. As seen in Figure 4*B*, HA-Fli1 (cotransfected with control gRNA) showed strong binding to MyD88 under basal unstimulated conditions (quantified and arbitrarily assigned as 100% binding in Fig. 4*C*), which was markedly reduced to roughly 20% upon IL1β stimulation for 30 min. We used TRB3 gRNA for knockdown of TRB3 in the same assay and found that, as expected, knockdown of TRB3 did not significantly impact the strength of basal, unstimulated Fli1–MyD88 interaction. However, upon IL1β stimulation, rather than an 80% drop in Fli1–MyD88 interaction, knockdown of TRB3 resulted in a only 40 to 45% drop in Fli1–MyD88 interaction (Fli1–MyD88 interaction quantified in Figure. 4*C*, corrected for expression levels of proteins). Unsurprisingly, coexpression of TRB3 severely abrogated both basal and IL1β-mediated Fli1–MyD88 interaction. Taken together, these data show that, in the absence of TRB3, Fli1 retains partial ability to sequester MyD88 and thereby attenuate IL1β-dependent signaling, while presence of TRB3 could augment IL1β signaling by overcoming Fli1-dependent brake on MyD88 recruitment.

### TRB3 enhances MyD88-dependent activation of NFκB reporter

In Figure 2 we showed that TRB3 gRNA is sufficient to blunt IL1β-dependent activation of NFκB-luciferase. Considering that the robustness of IL1R-driven signaling can be modulated by availability of MyD88, and that the presence of TRB3 creates permissive conditions for MyD88 recruitment to IL1β receptors, we asked whether overexpression of MyD88 was sufficient to activate NFκB-dependent transactivation and whether TRB3 could impact such an effect of MyD88. As shown in Figure 4*D*, overexpression of MyD88 robustly activated NFκB-luciferase activity (>10 fold), even in the absence of upstream receptor stimulation. While TRB3 alone did not significantly alter NFκB-luciferase activity, TRB3 significantly potentiated MyD88-dependent NFκB-luciferase activity increasing it from 10-fold activation to a 15- to 17-fold effect (compare dark blue bars from L-R in Fig. 4*D*). Interestingly, Fli1 coexpression (represented by hatched bars across the figure) did not impact MyD88-dependent NFκB-luciferase activity. These data are consistent with the fact that Fli1-mediated sequestration of MyD88 is supported by additional proteins or factors and the stoichiometry of endogenous interactions is most likely overcome by excess of expressed proteins in this artificial but informative assay. It is particularly noteworthy that, while Fli1 does not impact MyD88 activity under these conditions, Fli1 coexpression is sufficient to abrogate TRB3-mediated potentiation of MyD88-activated NFκB-luciferase activity. We interpret these data to suggest that the ability of TRB3 to potentiate IL1β/MyD88 signaling could depend directly on its ability to bind Fli1 rather than a complex of Fli1-associated factors.

### Fli1 knockdown and TRB3 overexpression augment stress-induced cell death

We have thus far seen that TRB3 influences inflammation at least in part by binding and disabling Fli1, which serves as a brake on inflammatory signaling in the absence of an appropriate stimulus. We therefore examined whether loss of Fli1 function or overexpression of TRB3 could induce cell death. To overcome the high expression of Fli1, we performed dual transfection of Fli1 siRNA in which cells are transfected with Fli1 or control siRNA for one passage (3–4 days) prior to and right before start of the actual experiment. Such a regimen was required for the significant knockdown of Fli1. As seen in Figure 4, *E* and G, Fli1 knockdown, and TRB3 overexpression, each had a low-grade impact on cleavage of caspase 3, prompting us to examine whether costimulation with a proinflammatory stress agent could unmask the apoptotic potential of both molecules. Although IL1β was the obvious choice as a stress agent, its potent apoptotic effect subsumed, rather than unmasked the proapoptotic potential of Fli1 knockdown and TRB3 overexpression. We therefore used TLR4-specific LPS as the costimulatory stress agent, treated cells for 48 h, and tested cellular extracts for cleavage of caspase 3, an indicator of end-stage cellular apoptosis. Interestingly, LPS treatment of Min6 cells even for 48 h showed negligible expression of cleaved caspase 3 (Fig. 4, *E* and *G*). However, LPS treatment served as an ideal unmasking agent for Fli1 knockdown/TRB3-induced apoptosis. These data are consistent with published results on the anti-inflammatory potential of Fli1 (29, 30) and proapoptotic impact of TRB3-induced (Fig. 2, *E* and *F*) iNOS protein (33, 34). Data presented in Figure 4, *E* and *G* are particularly noteworthy due to the striking similarity (see Fig. 4, *F* and *H*) between Fli1 knockdown and TRB3 overexpression to induce low-level cell death and the ability of LPS to augment apoptosis in both cases.

Finally, in Figure 4*I*, we present a schematic depiction for the potential mechanism by which TRB3 augments inflammatory signaling. The model also offers an explanation for resistance of TRB3KO islets to cytokine-induced beta cell death. We conclude that TRB3 and Fli1 have reciprocal roles in beta cell inflammation and in cytokine-induced cell death.

## Discussion

The role of TRB3 in ER stress has been extensively examined. However, its role in inflammation is less well documented. In this study we have examined the mechanism by which TRB3 supports inflammatory stress in beta cells. We have previously shown that IL1β and conditions mimicking insulitis induce TRB3 protein expression in both mouse and human islets (5, 10). IL1β activates upstream MAP3K MLK3, which rapidly binds and stabilizes TRB3, and together MLK3 and TRB3 stabilize the JNK signaling module for a full and robust inflammatory response (5). As a result, islets from TRB3 null mice show a roughly 50% decline in activation of the MLK3-JNK pathway (10). In addition to activation of MLK3-JNK, a full-blown inflammatory response in beta cells also requires activation of a second arm of signaling driven by the TAK1-IKK-NFκB pathway (15). In this study we report that similar to MLK3-JNK, phosphorylation and activation of the TAK1-IKK pathway is also attenuated in TRB3KO islets, resulting in partially suppressed NFκB-dependent gene expression and an overall dampening of the proinflammatory response.

At the mechanistic level we find that, unlike the MLK3-JNK module, TRB3 does not directly engage TAK1 or IKK, suggesting a more upstream role for TRB3. Here we have reported a novel interaction between TRB3 and Fli1, an immunomodulatory, actin-binding protein that controls the strength of postreceptor IL1β signaling. Specifically, Fli1 sequesters MyD88, a crucial and proximal adaptor that is recruited to the toll-like receptor (TLR) domain at the cytoplasmic end of the IL1β receptor (30). Upon receptor recruitment, MyD88 forms a complex with IRAKs1 and 4 (IL receptor associated kinase), triggering cross-phosphorylation of IRAKS, ultimately leading to TRAF6-mediated ubiquitination and activation of specific MAP3Ks including TAK1 (32). This study demonstrates that TRB3 is required for robust activation of both JNK and IKK underscoring the importance of TRB3 for two crucial proinflammatory pathways.

In addition to IL1β receptor, MyD88 also serves as a crucial proximal adaptor for the TLR4 receptor. Although its physiological ligand is not clear, the TLR4 receptor has been implicated in beta cell decline associated with type 2 diabetes. Interestingly, TRB3KO islets stimulated with LPS, a nonphysiologic ligand for beta cell TLR4, also show markedly attenuated IKK activation and a strong suppression of iNOS (Figs. S3 and S4) protein expression. Although a more thorough investigation is required, these early experiments open the door to the possibility that, in addition to IL1β, TRB3 may also be required for TLR4-dependent inflammation and, by extension, for beta cell inflammation associated with both T1D and T2D.

The TAK1-IKK-NFκB pathway is central to cellular inflammatory responses. A complete inhibition of NFκB would be expected to inhibit all target genes. However, our results show that loss of TRB3 attenuates but does not abolish IKK or JNK. Interestingly, such a decrease in peak activity and temporal activation of IKK-NFκB does not uniformly impact downstream gene expression targets (1, 35). Our findings are consistent with the discovery that modulation of amplitude and/or duration of kinase activation itself encodes key information for driving distinct gene expression programs. Complexity of promoters, coupled with cooperative gene activation by transcription factors that are networked and integrated with upstream signals, likely renders each gene uniquely responsive to kinetics of IKK activation. In the beta cell, key NFκB targets including iNOS, proinflammatory cytokines, and chemotaxis promoting protein MCP1 (macrophage chemoattractant protein)/CCL2 together activate a feed-forward inflammatory loop between islet resident macrophages and beta cells (36, 37). TRB3KO islets displayed a sharp decrease in MCP1/CCL2 gene expression and a comparatively modest decrease in iNOS gene activation. Surprisingly, we observed a small (less than 15–20%) decrease in MCP1 protein expression (not shown) but near-complete inhibition of iNOS protein expression in response to IL1β stimulation of TRB3KO islets. These data show that TRB3 may play an additional role in posttranscriptional regulation of cytokine-activated iNOS protein expression.

Depending on the cell type, iNOS-dependent nitric-oxide (NO) production is associated with effects ranging from increased survival to promotion of cell death. In cells where it has a deleterious role, NO generates powerful peroxynitrites, which augment oxidative stress and accelerate inflammation-induced cell damage (38, 39). A myriad of pleiotropic activities of NO have precluded a real consensus on the mechanism by which NO regulates cytokine-activated cell death (40, 41). Liu *et al.* (33) showed that, compared with control islets, primary islets from iNOS-knockout mice display 50 to 80% decrease in cytokine-induced necrosis, while Holohan *et al.* (34) showed that overexpression of iNOS alone is sufficient to induce beta cell apoptosis. We conclude that a strong inhibition of iNOS expression in TRB3KO islets may at least partially explain their resistance to cytokine-induced apoptosis.

TRB3 has been demonstrated to modulate kinase action by direct interaction with several kinases including mTORC1, AKT, MLK3, and MAP2Ks (3, 5, 9, 42). Here we identify TRB3–Fli1 interaction as a novel mechanism by which TRB3 augments beta cell inflammation and death. Fli1 can exert a synergistic or an antagonistic inflammatory role in a context-dependent manner (13, 43). Nevertheless, the rapidly expanding proinflammatory role of Fli1 lends credence to the ability of TRB3 to augment cytokine-dependent beta cell stress *via* Fli1. Results reported in this study suggest that TRB3 and Fli1 function in a reciprocal fashion. Fli1 and TRB3 behave like scaffold proteins and introduce a level of plasticity into the signaling process. Neither the gain of TRB3 function nor the loss of Fli1 by itself is sufficient to prompt rapid activation of cell death. In each case, additional low-grade stress was required to induce death suggesting that both Fli1 and TRB3 modulate rather than completely alter the dynamics of inflammation. Scaffold-to-scaffold interactions are increasingly being recognized as highly dynamic networks that control multiprotein complex formation. These transient interactions alter the temporal and kinetic flow of cellular information to elicit distinct biological and metabolic outcomes (44, 45, 46). Based on its ability to bind Fli1 and modulate the dynamics of receptor recruitment of MyD88, our results recast TRB3 as a protein that can modify flow of information to produce distinct biological outcomes.

## Experimental procedures

### TRB3 knockout mice

The mutant mouse strain was obtained from the European Mouse Mutant Archive (EMMA ID EM:02346), Helmholtz Zentrum Muenchen, Germany, as described (10). Mice were housed in a 12-h light/12-h dark cycle at controlled temperature (25 °C ± 1 deg. C). Genotyping was performed by PCR of genomic DNA according to the above-referenced protocols. In experimental procedures, we compared homozygous TRB3 knockout mice with wildtype littermates (control mice). TLR4KO mice were provided by Dr Richard Gallo (Department of Dermatology, UCSD). All experimental procedures were performed according to University of California, San Diego IACUC policies.

### Reagents

Antibodies used for Western blotting included anti-Myc, anti-Flag, anti-HA, anti-pJNK, anti-JNK, anti-pIKK, anti-TAK1, anti-pTAK1, cleaved caspase 3, and anti-β-tubulin from Cell Signaling. Anti-Fli1 and anti-iNOS were purchased from Santacruz Biotechnology, and anti-TRB3 antibodies were a gift from Dr Marc Montminy, Salk Institute. For immunofluorescence, mouse anti-BAX clone 6A7 (BD Bioscience), sheep anti-insulin (Binding Site), and *In Situ* Cell Death Detection Kit (Fluorescein) from Roche were purchased from commercial sources. Agarose-conjugated antibodies used for immunoprecipitations included Anti-HA agarose (Thermo Fisher Scientific) and Anti-FlagM2 agarose (Sigma-Aldrich). Streptavidin-Agarose was purchased from Pierce. Immunofluorescence and Western blotting employed fluorescent or HRP-conjugated secondary antibodies (Jackson ImmunoResearch), the latter detected using Supersignal West-Pico Plus chemiluminescence reagent (Thermo Fisher). Other reagents include IL-1β (Peprotech) and ultrapure TLR4-specific LPS (InvivoGen). Flag-TRB3 adenovirus was generated as described 3). Smartpool TARGETplus siRNA was used to knockdown Fli1, and nontargeting siRNA was used as control (Dharmacon-Horizon Discovery Sciences). TPCA-1 (Bio-Techne-Tocris) was used to inhibit the NFκB pathway, and MLK3 was inhibited using CEP11004 (gift from Cephalon Inc).

### Plasmids and constructs

HA-TRB3 and FLAG-β-tubulin constructs have been described elsewhere (5). Plasmids encoding HA-tagged LRR (leucine-rich-repeat) domains of LRRFIP2 (Addgene plasmid # 21152) and Fli1 (Addgene plasmid # 21151) were a gift from Dr Junying Yuan (Harvard University), and Flag-MyD88 (Addgene plasmid # 13093) was a gift from Dr Ruslan Medzhitov (HHMI, Yale School of Medicine). pcDNA3-HA-Fli1 was generated by PCR-based amplification of full-length, mouse Fli1, using pCMV-Sport6 mFli1 (Horizon Discovery) as template, and cloned into pcDNA3.1 vector using HindIII and Xho1 sites, in frame with a C-terminal HA tag. Myc-APEX2-TRB3 was generated by PCR amplification of APEX2 using pcDNA3-APEX2-NES as template (Addgene plasmid #49386, kind gift of Alice Ting, Stanford University) and subcloned into BamH1 and Xho1 sites of pcDNA3 Myc plasmid, followed by subcloning of TRB3 in frame with APEX2, using EcoRI and XbaI sites. NFκB-luciferase was purchased from Clontech. Nickase-Ninja TRB3 gRNA plasmids were purchased from ATUM Bioscience. Each plasmid expressed CMV promoter driving a Cas9N nickase mutant that causes single-strand breaks and coexpresses two gRNAs using dual U6 promoters. gRNA directed against GFP was included as control (47). Sequences of dual gRNAs targeting either Exon 2 or Exon 3 of the mouse TRB3 gene are included in Supporting information section.

### Primary islet isolation and Cell culture

Human islets were obtained from the Integrated Islet Distribution Program. Islets were handpicked and cultured overnight in CRML supplemented with 10% fetal bovine serum (FBS). Prior to experiments, islets were cultured in RPMI-1640, with 11 mM glucose and supplemented with 10% FBS. Mouse islets from WT and TRB3KO mice were isolated as described, using Liberase (0.2 mg/ml; Roche Applied Science) to distend the pancreas, and digested at 37 °C for 15 min. Preparations were washed with Hanks' buffered salt solution, and dissociated islets were purified using Histopaque (Sigma-Aldrich) gradients and cultured in RPMI 1640 medium supplemented with 10% FBS. Min6 cells (passages 15–18 *only*) were grown in Dulbecco’s modified Eagle’s medium containing 25 mM glucose supplemented with 4% heat-inactivated FBS and 50 μM β-mercaptoethanol.

### Transient transfections, nucleofection, and luciferase reporter assays

Transfections for reporter assays and co-IP experiments were carried out using Lipofectamine 2000 (Invitrogen) as per manufacturer’s instructions, and 40 to 48 h post transfection, cells were treated with 10 ng/ml IL-1β or 10 ng/ml TLR4-specific LPS as described. For gRNA experiments, 1 × 10^6^ Min6 cells were electroporated using Nucleofector Kit V (Amaxa GmbH) with 0.5-1 μg of plasmid DNA encoding TRB3-targeted gRNA (described above) and 0.4 μg of pcDNA3-RFP as nucleofection control and used for analyses 40 to 48 h post nucleofection. Luciferase assays were performed using CSH Protocols as described. For Gaussia luciferase, 10 to 30 μl of secreted medium was used with coelenterazine as substrate. Luciferase activity was measured using a tube luminometer (Berthold Technologies). For efficient knockdown of Fli1, a long-lived protein, dual siRNA transfections were performed using Dharmafect-3 reagent at a final concentration of 50 nm. Cells intended for Fli1 knockdown experiments were pretransfected with the respective siRNAs, split after 72 to 96 h, and retransfected with the same siRNAs before proceeding to conduct the experiments after a few days as described.

### RNA isolation and quantitative PCR

Islets isolated from WT and TRB3KO mice were stimulated with IL-1β for 2 and 4 h. Total RNA was isolated using RNAqueous isolation kit (Thermo Fisher) and mRNA reverse transcribed using PrimeScript RT Reagent Kit (Takara Bio USA). Quantitative PCR reactions were carried out using PowerUp SYBR-Green master mix using StepOne Real-Time PCR systems (Applied Biosystems). GAPDH mRNA levels were used for normalization. Relative mRNA expression was determined by the ΔΔCT method. Primers used for qPCR reactions will be provided on request.

### Proximity-dependent biotinylation of TRB3-interacting proteins

About 8 to 10 million Min6 cells were plated on 10-cm dishes and transfected with 8 ug of plasmid expressing the APEX2-TRB3 fusion protein. About 40 to 48 h post transfection, the cells were treated with 2 mM Biotin-tyramide (APEX-Bio Inc) for 2 h. Biotinylation was activated for exactly 1 min using a final concentration of 0.5 mM H_2_O_2_ and terminated using quenching solution as described (48). Biotinylated proteins in cellular lysates were pulled down using streptavidin-agarose, resolved on SDS-PAGE, and probed for interacting proteins using Western blotting.

### Splenocyte and islet coculture

Mouse splenocyte and islet coculture was performed as described (10, 18). Briefly, wildtype spleens were crushed and passed through a 70-μm mesh. Red blood cells were lysed in 0.15 M NH_4_Cl, and 1.5 × 10^6^ splenocytes per well were plated in 24-well dishes in RPMI supplemented with 10% heat-inactivated FBS. Splenocytes were stimulated with plate-bound anti-CD3 and exogenous anti-CD28 antibodies (10 μg/ml and 1 μg/ml, respectively; BD Bioscience) for 2 days. Isolated islets from WT or TRB3KO mice were rested overnight and cocultured using transwell filters in the presence of unstimulated or stimulated splenocytes. Human splenocyte and islet coculture was done using a similar protocol but slightly modified as described (18). For drug treatments, CEP11004 was provided by Cephalon Inc and TPCA1 and BAY-11-1085 were purchased from Tocris Inc.

### Immunofluorescence and TUNEL assays, microscopy, and image acquisition

Islets were fixed in Zinc buffered formalin (Anatech) for 1 h and processed for routine cryomicrotomy. TUNEL assay was carried out according to manufacturer’s instructions (Roche Diagnostics). Primary antibodies were visualized with species-specific secondary antibodies conjugated to fluorescent probes. Fluorescent images were acquired on a Zeiss 710 confocal microscope. All images were assembled in Photoshop CS6 (Adobe Systems Inc), and ImageJ (NIH). DAPI (1 μg/ml) was used as a nuclear counterstain.

### Protein–protein interactions and Coimmunoprecipitations

For identification of TRB3 interaction proteins, freshly split Min6 cells (passage 16) were infected with Flag-TRB3 adenovirus or HA-tagged TRB3 lentivirus and approximately 100 × 10^6^ cells in multiple batches of 10 to 12 × 10^6^ cells were allowed to form pseudoislet clusters with gentle shaking in the cell culture incubator, and the medium was replaced after 16 to 20 h. Cells/pseudoislets were pooled and harvested at 36 h, using standard co-IP conditions using NEHN buffer containing 150 mM NaCl, 0.5 mM EDTA, 20 mM Hepes pH 7.8, and 0.5% NP-40 (Pierce), with a full complement of protease and phosphatase inhibitors. Lysates were subjected to immunoprecipitation using precleared agarose-bound Flag or HA tag-specific antibodies for 4 h and eluates washed five times with gentle shaking, for 3 to 5 min, resolved using SDS-PAGE, stained using colloidal Coomassie stain, destained, specific bands excised and subjected to in-gel trypsinization followed by mass spectrometric analysis. GFP adenovirus or RFP lentivirus were used as control. The experiment was repeated using Min6 cells. Pseudoislets were used for co-IP experiments of IL1β-induced TRB3 binding to endogenous Fli1. Min6 monolayers were used for interaction of expressed proteins. For TRB3 interaction with Fli1 from human islets, Flag-tagged TRB3 was expressed in cultured cells, purified by immunoprecipitation using NEHN, the beads were washed with NEHN- twice with 300 mM NaCl concentration and twice with NEHN-150 mM NaCl.

### Sample preparation for mass spectrometry

Sample immunoprecipitates were extracted in guanidine solution, methanol precipitated, and the pellet was suspended in 8 M Urea-Tris buffer followed by 10 mM TCEP mM and 40 mM chloroacetamide solution, the urea concentration reduced to 2 M and used for tryptic digestion. Digested peptides were acidified (0.5% trichloroacetic acid) and desalted, and the concentration was measured using BCA Protein Assay Kit (Thermo Scientific Pierce). One microgram of protein was injected for each label-free quantification run.

### Mass spectrometry and identification of proteins

Trypsin-digested peptides were analyzed by ultrahigh pressure liquid chromatography coupled with tandem mass spectroscopy (LC-MS/MS) using nanospray ionization. The nanospray ionization experiments were performed using an Orbitrap fusion Lumos hybrid mass spectrometer (Thermo) interfaced with nanoscale reversed-phase ultrahigh pressure liquid chromatography (Thermo Dionex UltiMate 3000 RSLC nano System) using a 25-cm, 75-micron-ID glass capillary packed with 1.7 μm C18 (130) BEH beads (Waters Corporation). Peptides were eluted from the C18 column into the mass spectrometer using a linear gradient (5–80%) of acetonitrile (ACN) at a flow rate of 375 μl/min for 1 h. The buffers used to create the ACN gradient were Buffer A (98% H2O, 2% ACN, 0.1% formic acid) and Buffer B (100% ACN, 0.1% formic acid). Mass spectrometer parameters are as follows: an MS1 survey scan using the orbitrap detector (mass range [*m/z*]: 400 to 1500 using quadrupole isolation), 120,000 resolution setting, spray voltage of 2200 V, ion transfer tube temperature of 275 °C, AGC target of 400,000, and maximum injection time of 50 ms followed by data-dependent scans (top speed for most intense ions, with charge state set to only include +2–5 ions, and 5 s exclusion time, while selecting ions with minimal intensities of 50,000 at which the collision event was carried out in the high-energy collision cell (HCD Collision Energy of 30%), and the fragment masses were analyzed in the ion trap mass analyzer (with ion trap scan rate of turbo, first mass *m/z* was 100, AGC Target 5000 and maximum injection time of 35 ms). Mass spectrometry parameters were as described. Protein identification and label-free quantification were carried out using Peaks Studio 8.5 (Bioinformatics Solutions Inc).

### Statistics

Where applicable, area under the curve values were calculated using GraphPad Prism software. Differences between means were examined using analysis of variance (ANOVA) followed by a Bonferroni *post hoc* comparison. Analysis was also performed using GraphPad Prism software. Unless specified, data presented in bar graphs show average fold effects from three independent experiments. Within each experiment values were normalized to WT control, assigned a value of 1.

## Data availability

All data pertaining to the document are contained within the article. This study is focused on a single TRB3 interacting protein. Therefore, curated data on the TRB3 interactome is included in Table S1. Larger datasets related to the interactome will be made available upon request on a collaborative basis.

## Supporting information

This article contains supporting information.

## Conflict of interest

The authors declare that they have no conflicts of interest with the contents of this article.
