# Supplementary material for: Stress-induced pseudokinase TRB3 augments IL1β signaling by interacting with Flightless homolog 1
Source: J Biol Chem. 2023 May 11;299(8):104803. doi: 10.1016/j.jbc.2023.104803 (PMC10432976; doi:10.1016/j.jbc.2023.104803)
Supplement: Supporting Figures S1–S4 and Table S1 [file mmc2.pdf]

Supporting Information:

Figure S1)

TRB3 gRNA Exon 2

5′ GGCCCCAGTCGAGTTGCAGGGGCCACAGCA**GGTGACAAGTCTGAGGCGGG****AGG**TGG 3′
CCGG**GGT****CAGCTCAACGTCCCCGGTG**TCGTCCACTGTTCAGACTCCGCCCTCCACC

TRB3 gRNA Exon 3

5′ GTGCCACGGCACTAGCCATCTGCCGGAAGA**GCCCGGCAGCCTCGGACTCTG****GGA**TA 3′
CAC**GGT****GCCGTGATCGGTAGACGGCC**TTCTCGGGCCGTCGGAGCCTGAGACCCTAT

Figure S1: Sequence and configuration of TRB3 gRNA for Cas9 D10A Nickase based genome editing: Design of two independent gRNAs targeting Exon 2 (A) and Exon 3 (B) of the TRB3 gene. The PAM sequences are highlighted in red and two gRNA are highlighted in blue. Arrows indicate the orientation of the oligomer/gRNA is indicated by black arrows.

Figure S2:

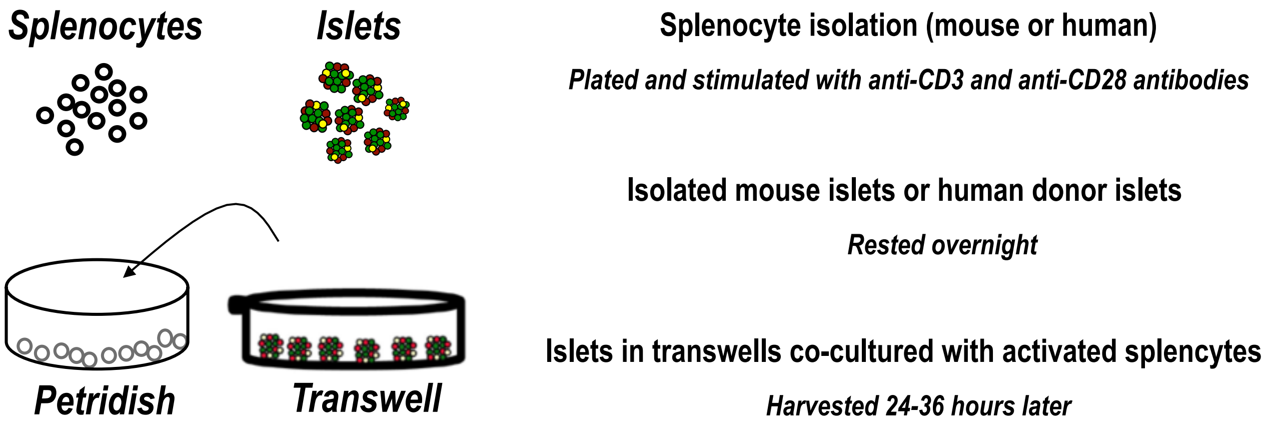

Figure S2: Schematic depicting Splenocyte-Islet co culture: Primary splenocytes from WT mice (or cadaveric human donor) were isolated on day 1 and plated as indicated to activate an immune response. Human donor islets or islets from WT and TRB3KO mice, were isolated the next day and allowed to rest and each set of islets was cocultured with activated or unstimulated splenocytes on day 3. For human splenocytes, cadaveric human spleen was used.

Table S1:

A

	Unstim	Stim 48 hrs	Unstim	Stim 48 hrs
**TNF-α**	3	46.6	167	4385
**IL-4**	3	137	12	1181
**IL-5**	3.5	68.3	7	345
**IL-6**	7.3	404	1825	4108
**IL-10**	6.99	333	97	9030
**RANTES**	27	191	150	8860
**GM-CSF**	3.2	370	219	5366
**IFN-γ**	4.75	10200	725	8690
**IL-1ß**	1.2	13.6	51	595

B

sample ID	Wells	GM-CSF pg/mL	IFN-g pg/mL	IL-10 pg/mL	IL-1b pg/mL	Il-6 pg/mL	MCP-1 pg/mL	MIP-1a pg/mL	Rantes pg/mL	TNF-a pg/mL
WT Stim 24hr	G8 H8	43.1	400	32.9	<3.2	179	28.3	485	35.3	20.4
WT Stim 48hr	A9 B9	1570	12000	685	3.87	973	149	9050	115	133
WT Stim 72hr	C9 D9	2610	12100	1760	4.55	1240	400	9200	142	145
WT unstim 24hr	C10 D10	<3.2	<1.25	7.94	<3.2	36.8	<3.2	71.2	23.7	1.91
WT unstim 48hr	E10 F10	9.12	<1.25	12.5	<3.2	57.2	<3.2	75.9	30.4	3.86
WT unstim 72hr	G10 H10	<3.2	<1.25	5.65	<3.2	50.1	<3.2	58.2	27.2	1.19

Table S1: Secretory profile of stimulated splenocytes: Medium secreted by unstimulated and stimulated splenocytes from human splenocytes (**A**) or mouse splenocytes (**B**) was profiled for the indicated cytokines by multiplex ELISA.

Figure S3:

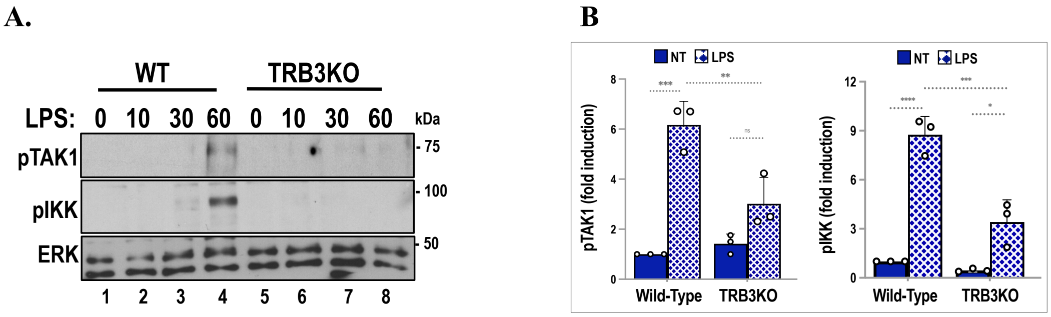

Figure S3: LPS-activated signaling is attenuated in TRB3KO islets. A) Wildtype (lanes 1 to 4) and TRB3KO (lanes 5 to 8) islets were treated with 5ng/ml of TLR4 receptor-specific LPS for the indicated times, and lysates were subjected to western blotting using anti-phosphoTAK1 and phospho-IKK antibodies. Total ERK protein levels are shown as control. **B)** Graph shows fold-peak activation of LPS-stimulated kinase (at 60 mins) normalized to unstimulated WT control signal density (** p<0.05, **<0.005, *** <0.0001*). Data shown represents densitometric analysis of three independent experiments, each using islets pooled from multiple mice.

Figure S4)

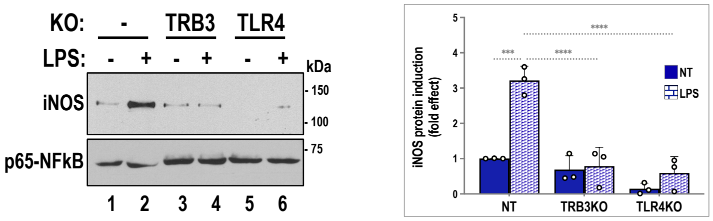

Figure S4) LPS-induced iNOS expression is attenuated in TRB3KO islets. Western blot for iNOS expression using protein extracts from pooled islets treated with 10 ng/ml LPS for 8 hours. Groups included are WT (lanes 1-2), TRB3KO (lanes 3-4) and TLR4KO mice. **B**) Quantification of iNOS protein was performed using densitometric analysis of three independent experiments, and represents average protein induction expressed as a fold effect of WT control islets. (**** *p<0.0001, *** p<0.001*
